# NNMT overexpression is an adverse prognostic factor in uterine leiomyosarcoma

**DOI:** 10.55730/1300-0144.5852

**Published:** 2024-04-16

**Authors:** Osman TÜRKMEN, Kamil Hakan MÜFTÜOĞLU, Nazmiye DİNÇER, Zeliha Esin ÇELİK, Serra AKAR İNAN

**Affiliations:** 1 Division of Gynecologic Oncology, Department of Obstetrics and Gynecology, Ankara Bilkent City Hospital, Ankara Yıldırım Beyazıt University, Ankara, Turkiye; 2 Department of Pathology, Ankara Bilkent City Hospital, Ankara, Turkiye; 3 Department of Pathology, Faculty of Medicine, Selçuk University, Konya, Turkiye; 4 Division of Gynecologic Oncology, Department of Obstetrics and Gynecology, Ankara Bilkent City Hospital, University of Health Sciences, Ankara Turkiye

**Keywords:** Nicotinamide N-methyltransferase, leiomyoma, leiomyosarcoma, survival, metastasis

## Abstract

**Background/aim:**

Uterine leiomyosarcomas (uLMS) are extremely rare high-grade tumors with a poor prognosis. Their etiopathogenesis remains largely unknown. The uterus is the most frequent site for LMS. uLMS and uterine leiomyoma (uLM) must frequently be differentiated in patients with a uterine mass. Nicotinamide N-methyltransferase (NNMT), a cytoplasmic protein, is involved in the progression and spread of a variety of cancer types. The expression of NNMT in a mesenchymal malignancy was not examined previously. This study represents the first investigation into NNMT expression in uLMS, uLM and benign uterine myometrium and correlates NNMT overexpression with worse prognosis in uLMS.

**Materials and methods:**

The expression of NNMT was investigated by immunohistochemistry on formalin-fixed paraffin-embedded tissue of uLMS in 31 patients, uLM in seven patients and benign myometrial in 31 patients.

**Results:**

The expression of NNMT in uLMS was markedly higher than in uLM and normal myometrial tissue (p < 0.001). The expression of NNMT in early stage uLMS was lower than in advanced stage disease (p = 0.034). NNMT expression was an independent prognostic factor in predicting recurrence-free survival in uLMS (p = 0.037).

**Conclusion:**

NNMT can aid in the preoperative differentiation of uLMS and uLM. The consequences of NNMT overexpression, such as the activation and inactivation of oncoproteins and tumor suppressor proteins, respectively, as well as the enrichment of the cancer stem cell population, overlap with the major mechanisms responsible for poor prognosis in mesenchymal tumors. NNMT may be investigated further in the context of antitumor treatment in patients with mesenchymal malignancies.

## 1. Introduction

Uterine sarcomas constitute 1% of gynecological malignancies and 3–7% of all uterine tumors [1]. Following the classification of uterine carcinosarcomas as metaplastic carcinomas, uterine leiomyosarcoma (uLMS) remains the leading histological subtype of all uterine sarcomas. uLMS is the most frequent site of LMS and is most commonly observed during perimenopause [2]. Most uLMS cases are high-grade tumors with unfavorable prognoses. As such, there is a need to elucidate the molecular pathways involved in their development and identify targets for antineoplastic treatment. Large-scale studies on these tumors are limited [3–6].

uLMS is usually diagnosed incidentally after histopathologic analysis of a myomectomy or hysterectomy specimen. Although both uLMS and uterine leiomyoma (uLM) arise from the smooth muscle cells of the uterine myometrium, they are regarded as distinct entities based on their different mutational and karyotypic origins [6]. uLMS and uLM, the most common benign gynecologic tumors [7], must be differentiated in patients with uterine masses, as the two are very different in terms of prognosis and management.

Nicotinamide N-methyltransferase (NNMT) is a cytoplasmic protein that is increasingly accepted as a protumorigenic enzyme involved in the progression, invasion, and spread of various cancer types. Its overexpression has been consistently observed in aggressive tumors and is correlated with worse prognosis [8–10]. NNMT overexpression leads to global DNA hypomethylation that subsequently renders oncogenic proteins active and the tumor suppressor proteins inactive [8–10]. The expression of NNMT in mesenchymal malignancies has not been examined. This appears to be the first study to investigate NNMT expression in uLMS. The expression levels of NNMT in uLMS, uLM, and benign uterine myometrium were compared, and NNMT overexpression correlated with disease prognosis in uLMS.

## 2. Materials and methods

### 2.1. Tissue samples

The study encompassed 69 patients who received treatment for uLMS or uLM at a tertiary referral center from 2010 to 2021. To conduct the research, formalin-fixed paraffin-embedded (FFPE) tissue was retrieved, consisting of 31 patients diagnosed with uLMS, seven patients with uLM, and 31 patients with benign uterine myometrial tissue. All samples were obtained from total abdominal hysterectomy specimens. uLM and benign myometrial samples were obtained from patients without a uLMS diagnosis.

Patients diagnosed with a malignancy or those who had received radiotherapy or chemotherapy before surgery were not included in the study. The necessary data including age, pathologic diagnoses, and stage based on the International Federation of Gynecology and Obstetrics (FIGO) for uLMS were obtained from patient records. The diagnosis of uLMS was made based on the 2020 WHO criteria [11], which considered factors such as the degree of histologic atypia, tumor cell necrosis, and increased mitotic rate. The staging of uLMS was determined using the 2009 classification of FIGO [12].

Patients diagnosed with uLMS underwent debulking surgery, which included total hysterectomy with bilateral salpingo-oophorectomy and resection of any visible metastasis either during their initial surgery or within one month after a definitive diagnosis of uLMS based on the myomectomy specimen. Following the surgery, the patients received appropriate adjuvant treatment based on their FIGO stage.

To assess the recurrence-free survival (RFS), the period was accepted as the time between the end of primary therapy and the time of diagnosis of recurrent disease. Approval from the Institutional Ethics Committee was obtained prior to the study (approval number: E2-22-2773).

### 2.2. Immunohistochemical analysis

Immunohistochemical analysis was conducted on FFPE tissue. The blocks were cut into 4-μm segments, followed by deparaffinization and rehydration. Subsequently, 10 mM of sodium citrate buffer (pH 6.0) was used with heat induction in the next phase of antigen recovery. Tissue peroxidase was blocked with hydrogen peroxide (3%), which was left for 15 min. Detection of NNMT expression was performed using a murine monoclonal antihuman antibody (diluted at 1:400) (NBP2-00537; Novus Biologicals, USA). Then the secondary antibody was added and diaminobenzidine was used for a 3-min chromogenic reaction. Two experienced pathologists independently evaluated immunoreactivity on each slide, blinded to the clinicopathological characteristics of the patients. The NNMT score for uLMS was calculated according to its reactivity within the tumoral component rather than the surrounding tissue. Staining intensity was assigned a value between 0 and 3 (0: none, 1: mild, 2: moderate, 3: strong), and this was multiplied by the percent of positive cells at 400× magnification.

### 2.3. Statistical analysis

For statistical analysis, Kruskal–Wallis and subsequent Mann–Whitney U comparisons with Bonferroni modifications were employed to compare NNMT scores among the three tissue groups. To predict adverse survival, NNMT score data were transformed into dichotomous data of “low” and “high” expression using receiver operator characteristic curve analyses with cut-off as well as sensitivity values. The relationship between NNMT reactivity scores and survival was investigated using Kaplan–Meier analysis, and the log-rank test for establishing statistical significance. Variables with a p-value of ≤ 0.2 on univariate analyses were entered into the Cox proportional hazards model. p < 0.05 was accepted as statistically significant. Statistical analysis was made with the SPSS statistical software package (version 27.0; IBM Corp., Armonk, NY, USA).

## 3. Results

**Figure 1:**
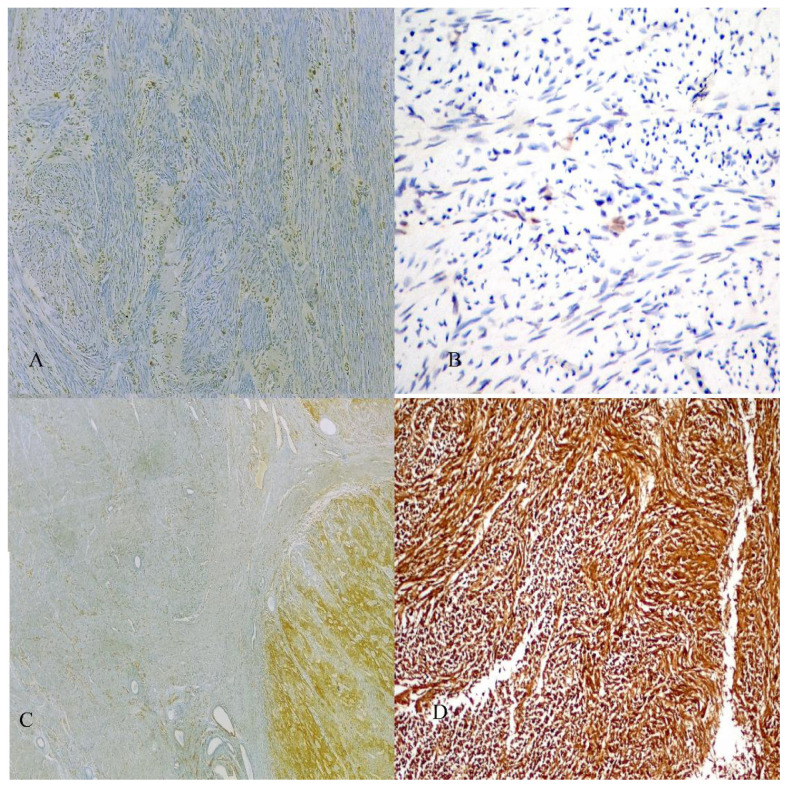
NNMT expression in A) benign uterine myometrium corresponding to an NNMT immunoreactivity score of 10 (original magnification ×200), B) uterine leiomyoma (uLM) corresponding to an NNMT immunoreactivity score of 5 (original magnification ×40), C) in uterine leiomyosarcoma (uLMS) adjacent to endometrial glands and tumor-free myometrium corresponding to an NNMT tumor score of 210 (original magnification ×200), D) in uLMS corresponding to an NNMT tumor score of 300 (original magnification ×10).

The mean age of the patients was 48.9 ± 10.6 (33–76). A total of 31 uLMS, 31 benign uterine myometrial tissues, and 7 uLM were analyzed for NNMT immunoreactivity. NNMT immunoreactivity was detected in all uLM and uLMS cases and in 17 (54.8%) benign myometrial tissue cases. NNMT immunoreactivity was cytoplasmic in all patients. NNMT staining scores for uLMS were obtained for the tumor. In terms of disease staging, 75% of patients with uLMS had early and 25% had advanced stage disease. Figure 1 presents illustrative examples of NNMT staining with corresponding NNMT scores. Figures 1A and 1B depict low NNMT reactivity scores of 10 and 5, respectively. Figures 1C and 1D demonstrate higher immunoreactivity scores of 210 and 300, respectively.

The mean staining scores are listed in Table 1. The average NNMT staining score for uLMS (148.2 ± 103.7) was significantly higher than that for uLM (23.6 ± 12.5) (p < 0.001) (Table 1). The mean NNMT score was significantly higher in uLMS than in benign myometrial tissue (28.3 ± 52.6) (p < 0.001). The mean NNMT score for early stage uLMS (137.4 ± 93.7) was lower than that for advanced stage disease (220.0 ± 115.3) (p = 0.034).

The median follow-up period for the patients was 60 (6–105) months. Out of the 31 patients, a metastatic recurrence was observed in 18 of them during this period.

**Figure 2:**
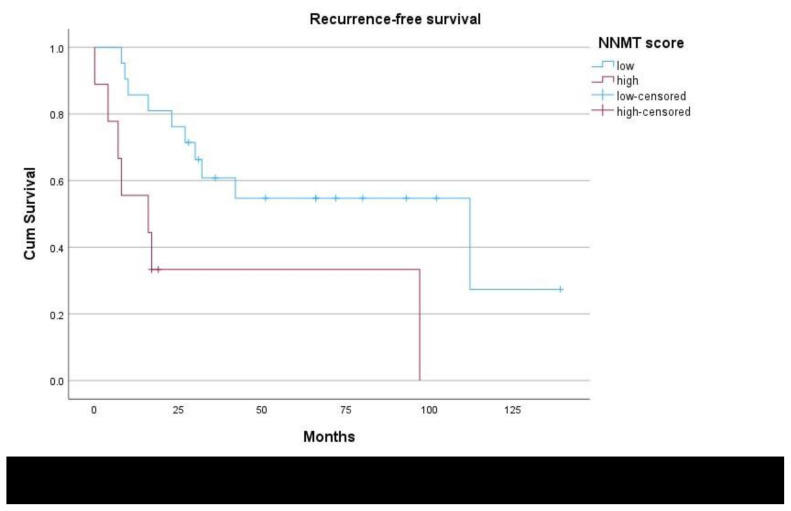
Recurrence-free survival (p < 0.001) in patients with “low” and “high” expression of nicotinamide N-methyltransferase (NNMT).

In the study a cut-off value of 200 was used for the NNMT tumor score to classify low and high expression levels of NNMT. Patients with low NNMT expression had a mean RFS of 79.0 months (95% CI: 53.9–104.0), whereas those with high NNMT expression had a mean RFS of 38.1 months (95% CI: 8.5–67.7), indicating a significant difference (p = 0.006) (see Table 2 and Figure 2). NNMT score, age and stage of disease were factors that were found to significantly affect RFS on univariate analyses (Table 2) and were entered into the multivariate analysis. Since all patients underwent complete cytoreduction followed by adjuvant therapy as necessary these factors were not included in the analysis. Moreover, in the multivariate analysis for recurrence-free survival, NNMT expression was found to be an independent prognostic factor (p = 0.037) (Table 2).

## 4. Discussion

Uterine leiomyosarcoma (uLMS) is an extremely rare tumor developing from the smooth muscle of the uterus. uLMS constitute 2–5% of all uterine malignancies [13]. uLMS has poor prognosis and is associated with early relapse and treatment failure. The inactivation of tumor suppressor proteins following the loss of RB1 and PTEN genes and inherited TP53 mutations increases the risk of uLMS development [14,15]. Additionally, radiation exposure and tamoxifen use have been associated with an increased incidence of uLMS [14]. However, the exact pathogenesis of uLMS is unknown. The mesenchymal expression of NNMT was investigated in this study.

NNMT expression in uLMS was significantly higher than that in benign uterine myometrium. This finding is consistent with the tumorigenic role of NNMT observed in different cancer types. Owing to its higher affinity for methyl groups, NNMT can outcompete other cellular methyltransferases in the consumption of cellular methyl molecules, leading to a state of global DNA hypomethylation. NNMT is thought to activate certain protooncogenes such as AKT and silence tumor suppressor proteins such as PP2A through DNA hypomethylation [8,9]. Initiation of the PI3K/AKT/mTOR cellular pathway is thought to be a consequence of loss of the PTEN gene and is the most common oncogenic pathway described in uLMS [14,15]. Additionally, sarcomas have been largely linked to aberrant p53 expression, and NNMT overexpression is significantly correlated with aberrant p53 staining patterns in endometrial [17] and breast cancers [18].

NNMT was found to be critical in the maintenance of the pluripotency of human embryonic stem cells through the suppression of the histone H3K27me3. The decrease in H3K27me3 due to NNMT overexpression appears to be pivotal for the sustenance of stem cell pluripotency [19]. The acquisition of stem cell properties induced by NNMT overexpression is another mechanism involved in treatment resistance and tumor relapse [19,20]. A study using a tumorigenic human mesenchymal stem cell model revealed that overexpression of the NNMT gene resulted in enhanced radiation resistance [20].

Another route by which NNMT overexpression leads to metastasis is via the initiation of epithelial-to-mesenchymal transition (EMT), that is the conversion of epithelial cell characteristics into those of mesenchymal cells. This process is a prerequisite for cancer cell invasion and spread [21,22]. A factor critical for EMT is the transforming growth factor-β1 (TGF-β1), which increases following NNMT overexpression [21,22]. Sarcomas may differ from carcinomas in that they do not require EMT to acquire metastatic capabilities because they are already endowed with these characteristics. These intrinsic phenotypes shared by sarcomas are thought to be responsible for the poor prognosis. However, although not completely known, EMT may also occur in mesenchymal tumors [23–27].

Although chromosomal instabilities (complex numerical and structural chromosomal aberrations) are considered representative of uterine smooth muscle tumors, none are considered diagnostic [16]. A cytogenetic or mutational link between uLMS and uLM has not yet been found [3-6]. The possibility of the presence of an undiagnosed uLMS within the uLM may alter the timing and route of management. In this study, average NNMT scores were significantly higher in uLMS than in uLM. uLMS are thought to arise de novo [16]; however, differentiating uLMS from uLM remains a challenge in any uterine mass, and there is a need for a marker that would aid in their preoperative discrimination.

NNMT expression in advanced stage uLMS was significantly higher than that in early disease. Previous studies, including those on gynecologic cancers, have demonstrated a significant correlation between NNMT overexpression and advanced disease stage, indicating an association between NNMT overexpression and cancer progression, invasion, and metastasis [17], [28–31]. Finally, NNMT overexpression was an independent prognostic factor along with disease stage for predicting recurrence-free survival. NNMT overexpression was also associated with shorter overall survival but was not found to be an independent predictor of overall survival, probably due to the limited sample size and fewer events in this study. Many studies have reported an inverse correlation between NNMT expression and patient survival. Recently, a metaanalysis of 13 retrospective studies on various carcinomas reported that elevated NNMT was associated with shorter disease-free and overall survival [32].

Although the number of patients included can be considered as one of the limitations of this study, the rarity of these tumors poses a challenge in conducting large-scale studies. Additionally, the lack of gene expression investigations in this study makes it difficult to elucidate the exact molecular pathways involved in NNMT-related tumorigenesis in uLMS.

Despite the limitations of this study, these findings can be extrapolated to extrauterine LMS. Specifically, a marker that can aid in the preoperative differentiation of uLMS and uLM could help reduce the number of unnecessary interventions and morbidity in patients presenting with a uterine mass. The consequences of NNMT overexpression, such as the activation and inactivation of oncoproteins and tumor suppressor proteins, respectively, as well as the enrichment of the cancer stem cell population overlap with the major mechanisms responsible for poor prognosis in mesenchymal tumors. NNMT may be further investigated as a potential target for anticancer therapy in patients with mesenchymal malignancies.

## Figures and Tables

**Table 1:** Comparison of NNMT scores between uterine leiomyoma (uLM), uterine leiomyosarcoma (uLMS) and uterine myometrium, as well as early and advanced-stage LMS.

	NNMT score (mean ± SD)	p
uLM	23.6 ± 12.5	<0.001*
Benign myometrium	28.3 ± 52.6	
uLMS	148.2 ± 103.7	
**uLMS stage**		
Early stage (I, II)	137.4 ± 93.7	**0.034**
Advanced stage (III, IV)	220.0 ± 115.3	

uLMS vs. myometrium (p < 0.001), uLMS vs. uLM (p < 0.001).

**Table 2:** Univariate and multivariate analyses of recurrence-free survival (RFS) for uterine leiomyosarcomas (uLMS) based on age, stage, and nicotinamide N-methyltransferase (NNMT) score.

RFS in months mean (95% CI)		Univariate analysis		Multivariate analysis	
		p	RFS hazard ratio (95% CI)	p	
Age	≤46	58.3 (29.6–87.1)	0.6		
	>46	43.8 (23.5–64.2)			
NNMT score	Low	79.0 (53.9–104.0)	**0.006**	3.5 (1.2–10.4)	**0.037**
	High	38.1 (8.5–67.7)			
Stage	I, II	77.1 (53.2–100.9)	**<0.001**	1.8 (1.1–2.6)	**0.010**
	III, IV	13.5 (4.8–22.2)			
